# Revisiting best practices: a reflection on the online evaluation and treatment of ADHD and implications for future practice

**DOI:** 10.1186/s13034-023-00593-z

**Published:** 2023-03-28

**Authors:** Max J. Rolison, Michael H. Bloch

**Affiliations:** 1 Yale Child Study Center, 230 South Frontage Road, New Haven, CT 06519 USA; 2 Department of Psychiatry, Yale University School of Medicine, New Haven, CT USA

## Context

Changes necessitated by COVID-19 have drastically changed how mental health services in the United States are provided and consumed. Many mental health encounters with therapists and prescribers are now conducted remotely and remain remote despite the diminishing health risks associated with COVID-19 infection. Patient accessibility, convenience, and comfort level with remote encounters rather than concerns about COVID-19 infection risk appear to be more significant drivers of this trend toward remote encounters. In addition, throughout the COVID-19 pandemic, there has been the proliferation of many online mental health platforms offering both therapy and medication management. The impending end of the Federal COVID-19 Public Health Emergency Declaration—and of the relaxed regulations governing the provision of remote mental health care—presents an opportunity for us to evaluate how mental health services, including psychiatric prescribing services, should be delivered going forward. A recent proposal from the Drug Enforcement Administration (DEA) suggests permanent modifications to rules for prescribing controlled substances via telemedicine—before prescribing stimulant medications, prescribers would need to either complete an initial in-person evaluation or have a referral from a provider who completed an in-person evaluation [1].

Attention deficit hyperactivity disorder (ADHD) diagnosis and treatment services have been central to the debate regarding providing mental healthcare online. In March 2020, in response to the COVID-19 pandemic, the Drug Enforcement Agency declared a public health emergency and suspended the in-person evaluation requirement associated with the Ryan Haight Act. The Ryan Haight Online Pharmacy Consumer Protection Act (2008) required at least one in-person evaluation before prescribing a controlled substance to prevent diversion and misuse [2]. However, due to the public health emergency, controlled substances could now be prescribed through telehealth without ever conducting an in-person evaluation [3]. This change opened new avenues for online services to provide ADHD care. Between 2020–2021, there was a 15% increase in Adderall prescriptions for adults aged 22–24, including through online services [4]. The popular press exposés have highlighted the shortcomings, pitfalls, and dangers of virtual ADHD treatment platforms that proliferated during the pandemic [5–7]﻿. The criticisms made in these articles, including substandard evaluation time for prescribers (typically less than 30 min) and external pressure on the employed doctors at the services to prescribe stimulant medications, paint a picture of these online platforms as virtual storefronts reminiscent of the opiate pill mills that developed in Florida during the oxycontin prescription boom [8] in the late 90 s and early 2000s.

## The importance of access

Despite the risks and pitfalls of online ADHD prescription services during the pandemic, it would be quite naïve and short-sighted to view all online mental health platforms as a significant public health hazard. Instead, it is essential to recognize the potential advantages of online platforms, including why they have become popular among patients and prescribers. Additionally, we must acknowledge the underlying market incentives that caused many less thorough and reputable online services to become the most prolific.

Untreated ADHD has significant health consequences. Roughly 10% of children in the US are diagnosed with ADHD, and a substantial portion will have symptoms that persist into adulthood [9–11]. Having ADHD is associated with a substantially increased risk of school dropout, arrest and incarceration, traffic citations and accidents, substance use disorders, and divorce. Virtually all of these risks have been demonstrated to be decreased when individuals are engaged in medication treatment for ADHD in epidemiologic studies [12]. Psychostimulant medications work more quickly and effectively than non-stimulant medication treatments for ADHD. It is also reasonably clear that medication management for ADHD is more effective than behavioral treatments for the core symptoms of ADHD [13].

## Advantages of online platforms

We should not overlook the potential for electronic ADHD treatment platforms to improve care and acknowledge several reputable platforms that have emerged during the pandemic. Online services offer the opportunity to provide accessible, high-quality, evidence-based ADHD care for adults and children. At this vital juncture, it is critical to consider best practices for assessing and treating ADHD, whether provided remotely or in person. For diagnosing ADHD [14], fundamental principles include clinical interviews with the patient and obtaining collateral (parents, teachers) about functioning across multiple settings (home, school, work, etc.). Evaluation should involve standardized measures of ADHD severity involving multiple informants rather than strictly subjective reports. Additionally, a thorough review of comorbid or alternate psychiatric disorders and a review of medical, social, and family histories are crucial to clarify ADHD symptoms caused by other etiologies. Treatment should involve psychoeducation; community, workplace, and school resources and accommodations; behavior therapy; and pharmacotherapy. Additionally, there should be frequent reassessment to evaluate for adequate treatment response, side effects of interventions, and whether continued treatment is necessary.

There are numerous advantages to online services. They can improve access to mental health care and make accessing assessment and treatment of psychiatric disorders easier, thereby addressing the nationwide provider shortage. In this way, online services can reduce the time waiting to connect with a provider, distance traveled, and inability to communicate due to conflicting schedules. Additionally, patients with psychiatric disorders may be more likely to seek treatment through an online service due to the stigma of psychiatric disorders. Furthermore, online services can also address financial barriers to accessing mental health care by providing subscription models that are less expensive for patients. Finally, although not implemented widely, online services may allow for more standardized and evidence-based assessment and treatment of patients using measurement-based care. Notably, telehealth and virtual care offer access to mental healthcare services in regions of the country where it may otherwise be difficult to obtain, such as in rural and frontier communities [15]. However, disparities remain in telehealth access in low-income and low-resource communities [16, 17]. The accompanying table outlines the basic tenants of quality evidence-based assessment of ADHD regardless of treatment setting (Table 1)

**Table 1:** Recommendations for assessment of ADHD

• Screening for Medical Conditions that can mimic the symptoms of ADHD—thyroid conditions, anemia, concussion, traumatic brain injury, sleep or vision problems
• Measurement of Physical Parameters at baseline that can be associated with side-effects of ADHD medication—heart rate, blood pressure, body weight, height
• Use Rating Scales to assess initial baseline symptom severity and monitor ADHD symptom change with treatment
• Screening and assessment for psychiatric conditions that can present with inattention symptoms—Depression or Bipolar Disorder, Anxiety, Psychosis, Substance use disorders, Trauma or PTSD, Learning Disorders, executive functioning difficulties, etc
• Checking of relevant controlled-substance prescription monitoring services to (1) examine past prescribed controlled substance use and (2) assess compliance with previous treatment
• Consideration of urine drug screening to (1) screen for recent substance use and (2) compliance with prescribed stimulant medications
• Use of collateral sources to verify childhood ADHD diagnosis and to assess the current level of impairment associated with ADHD symptoms

Pliszka S and AACAP Work Group on Quality Issues [14].

Using online platforms integrated into the electronic medical record offers the opportunity to increase the use of standardized assessment tools and structured clinical interviews that can better recognize other psychiatric contributors to inattention, such as self-report instruments such as the Development and Well-Being Assessment (DAWBA). Additionally, online platforms, such as meHealth (https://www.mehealth.com), can integrate the collection of standardized measure data from other sources, such as parents and teachers, to better understand the presenting symptoms. During treatment, online tools can continue to collect and track data from multiple informants to assess treatment efficacy. Furthermore, online platforms present the opportunity to integrate data about prior medication trials and tracking of prescription monitoring databases to identify possible abuse, diversion, or misuse. Checking prescription monitoring services is especially important as patients may give false information to obtain substances for intended misuse [18]. On the other hand, verifying that the person presenting before a provider is providing accurate identifying information can take time and effort. There are notable reports of online platforms providing treatment to patients under 18 without parental consent due to the limited capacity to verify age and identity [19]. Therefore, it is crucial to incorporate identification-verifying software and services to ensure that the intended patient receives the appropriate treatment. Regardless, the automatization, increased access, and economies of scale offered by online platforms also provide an opportunity to improve ADHD treatment for the better.

## Risks

Despite all these potential advantages of online treatment services, we must recognize the market incentives driving the widespread adoption of less thorough and scrupulous online ADHD prescription providers. The emphasis on clinical productivity (e.g., RVUs) and patient satisfaction rather than the quality of care delivered can lead to a consistent deviation from optimal care. Patient satisfaction is a problematic health metric, especially in ADHD treatment, when the patient may expect or demand psychostimulant treatment, and providing responsible care may often require otherwise. A compensation system focusing on clinical productivity and efficiency at the expense of quality financially rewards companies and prescribers engaging in these practices. These incentives encourage short encounter times, which may be insufficient, and the employment of prescribers willing to employ shortcuts in assessing and treating ADHD. These market incentives are not limited to online care delivery; they exist throughout healthcare. However, recently developed online platforms have followed suit in these market incentives rather than capitalize on the potential to offer a more robust treatment experience.

## Conclusion

In the table accompanying this article, we lay out the basic tenets of proper ADHD evaluations in children and adults regardless of treatment setting [14]. How many of those tenets are you currently following? Many negative consequences of online platforms are not unique to remote providers. However, they are instead a result of misaligned incentives in providing mental health care that is prevalent across contexts. The incentives encouraging the proliferation of substandard-online ADHD assessment and treatment services during the pandemic also influence the shape of mental health services at the local in-person level. As a profession and society, we should not simply outlaw the online prescribing of ADHD medication and not recognize the remarkable potential of these platforms to provide high-quality, evidence-based care if designed and implemented well.

## Data Availability

Not applicable.
